# Immunomodulatory Lectin-like Peptides for Fish Erythrocytes-Targeting as Potential Antiviral Drug Delivery Platforms

**DOI:** 10.3390/ijms222111821

**Published:** 2021-10-30

**Authors:** Maria Salvador-Mira, Veronica Chico, Monica Arostica, Fanny Guzmán, Nerea Roher, Luis Perez, Maria del Mar Ortega-Villaizan

**Affiliations:** 1 Instituto de Biología Molecular y Celular (IBMC), Universidad Miguel Hernández (IBMC-UMH), 03202 Elche, Spain; maria.salvador04@goumh.umh.es (M.S.-M.); vchico@umh.es (V.C.); luis.perez@umh.es (L.P.); 2 Instituto de Investigación, Desarrollo e Innovación en Biotecnología Sanitaria de Elche (IDiBE), Universidad Miguel Hernández (IDiBE-UMH), 03202 Elche, Spain; 3 Núcleo Biotecnológico de Curauma (NBC), Pontificia Universidad Católica de Valparaíso, Valparaíso 2373223, Chile; monica.arostica@gmail.com (M.A.); fanny.guzman@pucv.cl (F.G.); 4 Department Biologia Cellular, Fisiologia Animal i Immunologia, Institut de Biotecnologia i de Biomedicina (IBB), Universitat Autònoma de Barcelona (UAB), 08193 Cerdanyola del Vallès, Spain; nerea.roher@uab.cat

**Keywords:** peptide, red blood cells, erythrocyte, drug delivery, ligand, prophylactic, therapeutic, cell-targeting, lectin, fish

## Abstract

One of the challenges of science in disease prevention is optimizing drug and vaccine delivery. Until now, many strategies have been employed in this sector, but most are quite complex and labile. To overcome these limitations, great efforts are directed to coupling drugs to carriers, either of natural or synthetic origin. Among the most studied cell carriers are antigen-presenting cells (APCs), however, red blood cells (RBCs) are positioned as attractive carriers in drug delivery due to their abundance and availability in the body. Furthermore, fish RBCs have a nucleus and have been shown to have a strong involvement in modulating the immune response. In this study, we evaluated the binding of three peptides to rainbow trout RBCs, two lectin-like peptides and another derived from *Plasmodium falciparum* membrane protein, in order to take advantage of this peptide-RBCs binding to generate tools to improve the specificity, efficacy, immunostimulatory effect, and safety of the antiviral therapeutic or prophylactic administration systems currently used.

## 1. Introduction

In recent years, a major challenge in the design, delivery, and formulation of drugs and vaccines has been the development of more effective and safer delivery systems capable of enhancing immune responses. The ultimate goal of a vaccine is to induce a memory immune response, a process that is well-orchestrated by antigen-presenting cells (APCs), which acquire antigens from their environment, process them, and present them to T and B lymphocytes [1]. Many immunization strategies based on antigen delivery to APCs have been directed to manipulate immune responses to facilitate antigen uptake, by means of using chemokines or cytokines for APC recruitment [1,2], or even fusing antibodies or ligands to antigens to target specific receptors on the surface of APCs [3,4]. Despite this, there are certain limitations to consider such as the low percentage of APCs in the peripheral blood or the high cost and the difficulty in isolating and generating autologous APCs. To overcome these drawbacks, great efforts are directed to the search for long-circulating drug carriers/vehicles that can provide sustained release of circulating therapeutic agents and improve the specificity, efficacy, and safety of therapeutic agents [5,6,7]. In this regard, the idea of using red blood cells (RBCs) as natural and biocompatible carriers for the delivery and distribution of drugs or vaccines has been extensively studied [8,9]. Due to the large number of RBCs present in the organism and the wide diffusion throughout the body, the possibility of using them for the delivery of therapeutic and prophylactic agents is promising. Furthermore, compared to other drug delivery systems, RBCs are the champions for their unique longevity in the bloodstream and their biocompatibility [8]. Additionally, using RBCs as vaccine or immunostimulant platforms also avoids safety issues related to viral or bacterial vectors [9].

In order to target a treatment to a specific cell population or to make the treatments more specific, several studies have been directed to identify future molecular targets for drug delivery and generation of new vaccines, using RBCs from humans [10,11,12,13] or other vertebrate species as cell targets [14,15]. Most prophylactic delivery systems have demonstrated high immunogenic potency, which can cause non-specific stimulation of the host immune system [16,17,18]. Therefore, using RBCs as autologous carrier cells is of great importance in the therapeutic field by targeting drugs to the surface of these cells for adhesion or internalization in vivo, as well as serving as a vehicle to target antigens to APCs to enhance and boost immunity. In fact, RBCs have been tested as antigen carriers to prevent the production of specific anti-drug antibodies after repeated administration of therapeutic proteins by inducing specific immune tolerance [19,20]. Additionally, recently, it has been shown that mouse RBCs can act as artificial APCs by anchoring characteristic APC molecules to the cell surface so that these modified RBCs are able to activate T cells and promote the secretion of inflammatory cytokines [21]. Another approach investigated to develop novel therapies is the use of nanoparticles attached to RBCs as drug delivery systems, where the binding of nanoparticles to RBCs could prolong the residence time in the blood and minimize the rapid clearance of encapsulated therapeutic agents from the circulation [22,23].

On the other hand, it is important to emphasize that, unlike mammals, the RBCs of lower vertebrates, such as fish, birds, amphibians, and reptiles, preserve the nucleus and possess organelles within the cytoplasm [24], which provides them with the intracellular machinery necessary to become mediators of the immune response. Recently, nucleated fish RBCs have been shown to play an active role in the response against viral infections [25,26,27,28], in phagocytosis [29], and in antigen processing and presentation via the major histocompatibility complex (MHC) classes I and II [28,30,31,32,33,34]. Moreover, RBCs develop specific responses to different pathogen-associated molecular patterns (PAMPS) [35], overexpression of toll-like receptors (TLRs) [36], and are involved in the production of interferon, inflammatory cytokines, and chemokines [27,30,31,32,37,38]. Within the field of rational design of new prophylactic treatments, nucleated rainbow trout RBCs have been shown to express the antigen encoded by a DNA vaccine and respond to a DNA vaccine immunization inducing exogenous peptide presentation processes [31,39]. In addition, rainbow trout RBCs have been shown to be capable to endocytose bacterial inclusion bodies made of nanostructured recombinant antigenic proteins and modulate the expression of immune system genes [30]. In view of all these characteristics and due to their ability to generate and modulate the immune response, it is of interest to use nucleated RBCs as therapeutic or prophylactics targeting and delivery systems.

In relation to cell targeting, cell-binding ligands are becoming increasingly important, and their therapeutic applications have been growing. Cell type-specific ligands are powerful tools for functionalized therapeutic or prophylactic treatments since they can be easily incorporated into drug delivery platforms. Previous studies that combine protein therapeutics with ligands targeting RBCs have shown significant improvement in drug half-life and uptake in the target tissue [8,40,41].

In this article, we will focus on evaluating and analyzing the binding of three synthetic peptides to rainbow trout RBCs for their potential use in the development of new therapeutic or prophylactic delivery methods that can specifically target RBCs. Many of the cell-binding peptides that recognize receptors in various cell types have been identified by using peptide libraries [40,41,42]. Among them, different peptides have been found in the literature to target RBCs [43,44,45,46].

Among the identified peptides in the literature, we selected three peptides based on their reported affinity to bind nucleated RBCs or mammalian RBCs. One of the selected peptides is a highly active binding peptide derived from an integral membrane protein of *Plasmodium falciparum* (Pf25-IMP), which has been shown to bind strongly to human RBCs and block the entrance of the parasite *Plasmodium falciparum* into mammalian RBCs [43]. This peptide will be named in this article as 4341. Another selected peptide is a small peptide with lectin-like behavior, called odorranalectin (hereafter referred to as peptide 4342), which has been found to bind rabbit and human RBCs and has been described as a promising peptide for drug delivery [44]. Finally, the third selected peptide (hereafter referred to as peptide 4343) is a 6-amino acid lectin-like peptide derived from the S2 subunit of pertussis toxin with a high binding affinity to glycoconjugates containing sialic acid [45,46], and which has been described to bind goose nucleated RBCs [46]. In general, lectins are proteins or glycoproteins of non-immune origin that are ubiquitously distributed in plants, fungi, and animals and bind to sugar residues in the cell membrane and their binding is usually fast and strong [47]. Lectins have been attributed multiple significant biological functions, such as antiviral activities [47]. Their potential application for drug delivery and targeting has been well studied [44]. However, their participation in the modulation of the antiviral immune response has been scarcely studied.

Briefly, our results show that two of these three peptides, peptides 4342 and 4343, bind to rainbow trout RBCs and they are good candidates for linking up to new delivery systems and enhancing the antiviral immune response during treatment.

## 2. Results

### 2.1. In Vitro Evaluation of Peptide Binding to Rainbow Trout RBCs

**Figure 1:**
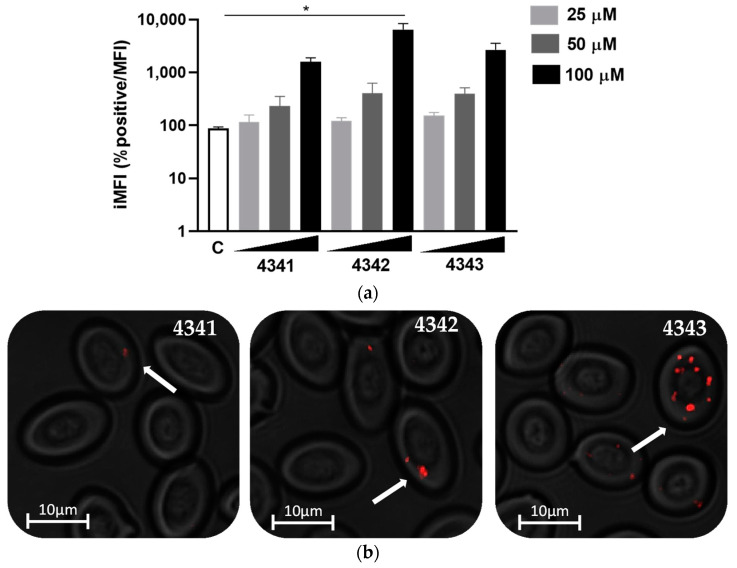
Peptide binding to rainbow trout RBCs in vitro, evaluated by means of flow cytometry. (**a**) Integrated median fluorescence intensity (iMFI) (% positive cells × MFI) of rainbow trout RBCs treated with different peptide concentrations, 90 min after incubation, analyzed by flow cytometry. Data represent the mean ± standard deviation (SD), *n* = 3. Kruskal–Wallis test with Dunn’s multiple comparison test was performed (* *p* < 0.05). C means untreated control RBCs. (**b**) Representative images of rainbow trout RBCs treated with the three selected peptides, 90 min after incubation, with 100 µM of each peptide. Images were taken with the IN Cell Analyzer 6000 Cell Imaging system at 40× magnification. Peptides are labeled with Rhd B (red). White arrows indicate peptide binding to RBCs.

**Figure 2:**
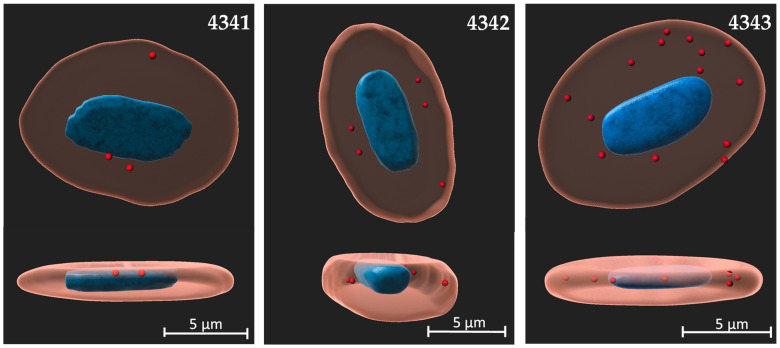
Volume 3D reconstruction of the visualization of the peptides binding to rainbow trout RBCs. Rhd-labeled peptides are represented in red, nuclei were stained with Hoescht and are represented in blue, and cell membrane was stained using CellMask Green and is represented in beige. The images were taken at 63× magnification. Confocal images were processed using the ZEN 3.2 (Blue edition). Volume rendering was performed with IMARIS v9.3. Upper images represent plan view and lower images represent perspective view.

In order to evaluate the binding of the peptides for RBCs, we carried out a dose-response assay using a concentration range from 25 to 100 µM. The binding of the Rhodamine (Rhd)-labeled peptides was evaluated by means of flow cytometry and calculated as the integrated median fluorescence intensity (iMFI), which is computed by multiplying the relative frequency (% positive) of cells with the median fluorescence intensity (MFI) of that population. The minimum concentration showed very low binding to RBCs, and concentrations of 50 and 100 µM showed higher and specific binding for all the peptides (Figure 1a,b). On the other hand, peptide 4342 showed the highest binding at 100 µM, followed by peptides 4343 and 4341 (Figure 1a). Besides, hemagglutination was not observed at the concentrations of peptides used (Supplementary Figure S1). Cell toxicity was not observed neither in vitro nor in vivo at the maximum concentration of peptides used (Supplementary Figure S2). Additionally, peptide-RBC binding was confirmed by confocal microscopy (Figure 2) which showed the presence of all the peptides inside the cell, although it was higher for peptides 4343 and 4342.

Moreover, an in silico evaluation of the peptides’ physicochemical descriptors was carried out. Dipole moment, number of hydrogen bond donors and acceptors, and logP of each peptide were calculated (Table 1). The results showed that peptide 4341 has a much larger dipole moment compared to 4342 and 4343. Negative logP values indicated that all peptides are hydrophilic. On the other hand, the three peptides showed close hydrogen bond donor/acceptor values (Table 1, Supplementary Figure S3). Additionally, the surface charge representations showed higher charge density on the left side structure than on the rotated 180° right side structure (Supplementary Figure S4). Both results indicated the peptides’ amphipathic capacity.

### 2.2. In Vitro Analysis of Gene Expression in Rainbow Trout RBCs after Peptide Treatment

**Figure 3:**
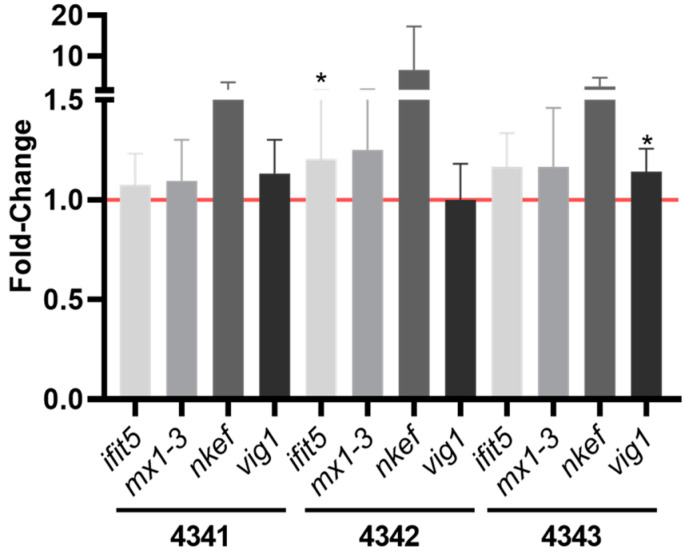
Antiviral immunomodulation of the peptides in rainbow trout RBCs. Antiviral gene (*ifit5*, *mx1-3*, *nkef*, and *vig1*) expression quantified by RT-qPCR in RBCs at 6 h post-treatment (hpt) with 50 µM of each peptide. Gene expression was normalized against *ef1α* and relativized to control cells (RBCs untreated) (red line). Data represent the mean ± standard deviation (SD), *n* = 4. Mann–Whitney test was performed (* *p* < 0.05).

We next investigated whether the treatment with the three Rhd-labeled peptides could generate an antiviral immune response in vitro in rainbow trout RBCs, by means of examining the expression profile of some genes characteristic of fish related to antiviral activity, such as *ifit5*: interferon-induced protein with tetratricopeptide repeats 5, *mx1-3*: interferon-inducible Mx, *nkef*: natural killer enhancement factor-like protein and *vig1*: viperin) by means RT-qPCR (Figure 3). The results showed that *ifit5* was statistically significantly upregulated by peptide 4342 and *vig1* by peptide 4343. On the other hand, it should be noted that slight and not statistically significant upregulation of *mx1-3* gene expression was induced by peptide 4342. Further, *nkef* gene expression was upregulated by all the peptides, and especially by peptide 4342, although not statistically significantly.

### 2.3. In Vivo Evaluation of Peptide Binding to Rainbow Trout RBCs

**Figure 4:**
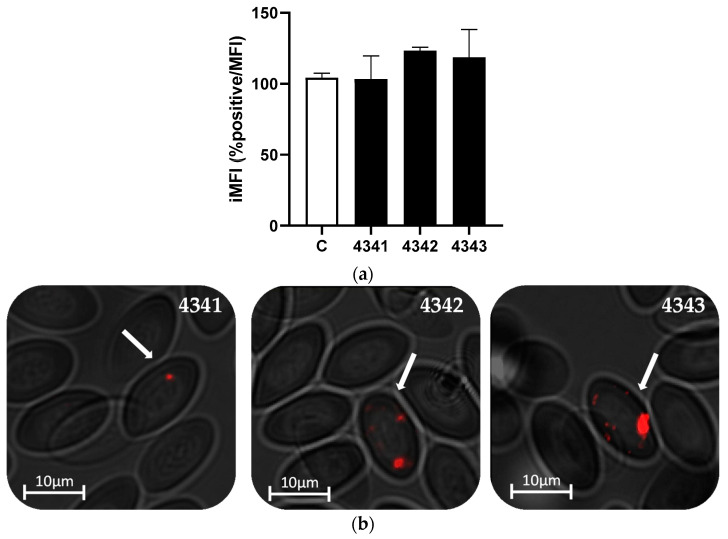
Peptide binding to rainbow trout RBCs in vivo, by means of flow cytometry. (**a**) Integrated median fluorescence intensity (iMFI) (% positive cells × MFI) of RBCs from blood of rainbow trout injected intravenously with the three selected peptides, 3 hpi with 100 µM of each peptide, analyzed by flow cytometry. Data represent the mean ± standard deviation (SD), *n* = 3. Kruskal–Wallis test with Dunn’s multiple comparison test was performed (*p* < 0.05). C means control RBCs. (**b**) Representative images of RBCs from blood of rainbow trout injected intravenously with the three selected peptides, 3 hpi with 100 µM of each peptide. The images were taken with the IN Cell Analyzer 6000 Cell Imaging system at 40× magnification. Peptides are labeled with Rhd (red). White arrows indicate peptide binding to RBCs.

In order to track in vivo the binding of the peptides to rainbow trout RBCs, we examined the peripheral blood of rainbow trout individuals injected with the peptides. At 3 h post-injection (hpi), blood was analyzed by means of flow cytometry and fluorescent microscopy. Flow cytometry evaluation showed the highest iMFI for peptide 4342, followed by peptides 4343 and 4341 (Figure 4a). Moreover, by means of fluorescence microscopy, we could verify the presence of peptides in RBCs (Figure 4b), although the number of cells showing bound peptides was lower compared to the in vitro evaluation, as shown by the iMFI (Figure 1a and Figure 4a).

## 3. Discussion

The biological barriers between the site of drug action and drug delivery are becoming increasingly important for the development of effective and safe therapeutic or prophylactics administration methods. The traditional approach is based on the use of vaccines as the most widely used method for the control of infectious and non-infectious diseases since they induce both humoral and cell-mediated immune responses [48]. Despite this, it is necessary to solve certain problems related to the efficacy and safety of vaccines, as well as the optimization of administration using low-cost and less aggressive methods. It is desirable to explore alternative molecules or a combination of them that can target specific cellular responses to confer protection and decrease possible side effects. For this reason, incorporating cell-binding ligands into drug delivery platforms has attracted special interest.

There is a great shortage of vaccines and prophylactic methods against diseases in fish farming which demonstrates the need to identify new strategies. Furthermore, many characteristics of the immune system of fish are still unknown. Nucleated RBCs from fish are postulated as promising cells due to their important role in the immune response [25,28,37,39]. On top of that it is the fact that RBCs are the most abundant cells in the bloodstream, which makes them ideal candidates for the development of new vaccination strategies to functioning as drug carriers in circulation.

Combining this evidence and trying to improve current protection strategies by making them more specific to RBCs, we tested and evaluated the binding of three synthetic peptides to RBCs, in vitro and in vivo. The selection of these peptides was based on an exhaustive bibliographic search that provided us with enough information to study them in our target cells considering that the three peptides have been tested in RBCs of humans and mice [43,44,45], and goose nucleated RBCs [46].

Our results demonstrated the binding of Rhd-labeled peptides to rainbow trout RBCs, with a higher affinity of the peptides 4342 and 4343 in vitro. Moreover, hemagglutination was not observed at the concentrations of peptides used. Both peptides have been shown to be involved in the lectin-like interactions [44,45,46]. Lectins are well-known and widely distributed proteins that bind to sugar residues in the cell membrane quickly and strongly because their binding involves many binding sites determined by a specific sugar code [49,50]. Furthermore, previous research has shown that lectins function as recognition molecules in cell–cell and cell–molecule interactions in many biological systems, making them excellent candidates for drug delivery and targeting [49,50].

In addition, our selected peptides were mainly found inside RBCs, instead of at the surface of the RBCs, as observed by confocal microscopy. In this regard, while negative logP values and dipolar moments indicated that structures have polar characteristics, especially peptide 4341, hydrogen bond donor/acceptors and surface charge representations indicated that peptides have amphipathic capacity, with hydrophobic and polar faces, which is typical of cell-penetrating peptides. This effect was more evident for peptides 4342 and 4343, which showed enhanced cell-penetrating activity. In this context, cell-penetrating peptides, which are particularly attractive peptides for vaccine delivery systems, have been widely used to enhance the transport of drugs of a wide range of sizes into the cells [51]. Among lectin-like peptides, the odorranalectin peptide, which is peptide 4342 in this study, has been shown to exhibit the potential of cell-penetrating peptides for targeted oligonucleotide delivery to glioblastomas across the biological barriers, with high stability in circulation [52]. The in vivo evaluation results also showed that peptides bonded to blood RBCs, although the number of RBCs with bonded peptides was very low, a result which is positive when administering a vaccine directed at RBCs since a greater binding could trigger problems at the level of the organism, like anemia.

The use of adjuvants has become a key method for increasing the potency of vaccines and drugs. Interestingly, it has been documented that short peptides or ligands exert immunomodulatory effects that can be used for the control of infectious and non-infectious diseases. For example, a short antimicrobial peptide, CM11, has been shown to upregulate key genes such as interleukin 1β (*il1β*), interleukin 8 (*il8*), or tumor necrosis factor α (*tnfα*), boosting the immune system of zebrafish against bacterial infections [53]. The muramyl dipeptide (MDP), a peptidoglycan muropeptide that consists of N-Acetylmuramic acid (MurNAc) and two amino acids [54,55] has been shown to induce a humoral and cellular response against viruses [56] and to improve the efficacy of a vaccine [57]. All these correlations lead to the use of short peptides as adjuvants to enhance a strong immune response. However, the ability of lectin-like peptides as modulators of the antiviral immune response has been scarcely studied. Lectins such as Concanavalin A or Wheat Germ Agglutinin are known to bind and activate lymphocyte receptors and induce cytokine gene expression and protein synthesis [58,59]. On the other hand, mannan-binding lectin (MBL) has been documented to play a key role in the lectin pathway of complement activation and to influence cytokine expression by modulating lipopolysaccharide (LPS)/Toll-like Receptor (TLR) signaling pathways [60]. Our results showed that mainly peptides 4342 and 4343 were capable of modulating the expression of genes related to the antiviral immune response, such as *ifit5*, *mx1-3*, *vig1,* or *nkef*. However, the mechanisms or pathways triggered by these lectin-like peptides remain to be studied. Apart from that, peptide 4341, in comparison to lectin-like peptides 4342 and 4343, showed lower induction of interferon-stimulated antiviral genes *ifit5*, *mx1-3*, and *vig1*. The interferon signaling pathway has been reported to be induced by *Plasmodium* ligands in mammalian cell types [61]. However, 4341 *Plasmodium*-derived peptide was not able to modulate interferon related genes in rainbow trout RBCs.

In summary, we identified two candidate peptides with immunomodulation capabilities for future vaccine delivery studies. The next step will be to attach the selected peptides to therapeutic or prophylactic tools targeted to RBCs, for the prevention of diseases with high impact on aquaculture.

## 4. Materials and Methods

### 4.1. Peptide Synthesis and Characterization

The three selected peptides (Table 2) were synthesized at the laboratory of Dr. Fanny Guzman. The peptides were synthetized by Fmoc solid-phase methodology using Rink Amide resin (0.65 meq/g) (Iris Biotech GmbH, Marktredwitz, Germany) and Fmoc amino acids (Iris Biotech GmbH) in a Liberty Blue automated microwave peptide synthesizer (CEM Corp., Matthews, NC, USA). Fmoc groups removal was completed with 20% *v/v* of 4-methyl piperidine (4MP) (Merck KGaA, Darmstadt, Germany) in *N*,*N*-dimethylformamide (DMF) (Merck) [62]. Furthermore, the coupling of Rhd B, for its later use in microscopy, was carried out manually using 5:5:5:7.5 equivalents of Rhd B:(2-(1H-benzotriazol-1-yl)-1,1,3,3-tetramethyluronium hexafluorophosphate (HBTU) (Iris Biotech GmbH): Oxyme-Pure (Iris Biotech GmbH): N-ethyldiisopropylamine (DIEA) (Merck) [63]. Peptide cleavage was performed with a solution of 92.5% trifluoroacetic acid (TFA)/2.5% triisopropylsilane (TIS)/2.5% diethanethiol (DOT)/2.5% ultrapure water), washed with cold ether and finally purified by C18 extraction columns with acetonitrile/water gradient to a purity higher than 95%. C18 columns and reagents were acquired from Merck. Peptides were characterized by HPLC and mass spectrometry (Water Corp., Milford, MA, USA) (Jasco and Shimadzu 2020) [64].

### 4.2. Animals

Juvenile rainbow trout (*Oncorhynchus mykiss*) were obtained from a commercial fish farm (Mundova, Río Mundo SLU Fish Farm, Albacete, Spain). Fish were maintained at the University Miguel Hernandez (UMH) facilities in a recirculating, dechlorinated, and progressively microfiltered water system, at 14 °C, and fed daily with a commercial diet (Skretting, Burgos, Spain). Prior to experiments, fish were acclimatized to laboratory conditions for 2 weeks. The number of individuals used is indicated for each experiment/figure. All activities involving animal handling and animal care were undertaken following the Animal Welfare Body, the Research Ethics Committee at the UMH, and the competent authority of the Regional Ministry of Presidency and Agriculture, Fisheries, Food and Water supply reviewed and approved all experimental protocols, according to Spanish [Real Decreto 1201/2005] regulations. The methodology was performed in accordance with the Spanish [Real Decreto RD 53/2013] and EU [EU Directive 2010/63/EU] regulations and recommendations for animal experimentation and other scientific purposes. The permit number of the Ethics committee is UMH.IBM.MOR.01.14-3.

### 4.3. RBCs Purification and In Vitro Treatment with the Peptides

Rainbow trout were sacrificed by overexposure to tricaine methanesulfonate (Sigma-Aldrich, Madrid, Spain) at 0.2 g/L. Peripheral blood was collected from the caudal vein using insulin syringes (NIPRO, Bridgewater, NJ, USA). Approximately 100 µL of blood was diluted in RPMI-1640 medium (Dutch modification) (Gibco, Thermo Fischer Scientific Inc., Carlsbad, CA, USA) supplemented with 10% fetal bovine serum (FBS) (Cultek, Madrid, Spain), 2 mM L-glutamine (Gibco), 1 mM pyruvate (Gibco), 2 µg/mL fungizone (Gibco), 50 µg/mL gentamicin (Gibco), and 100 U/mL penicillin/streptomycin (Sigma-Aldrich). Then, RBCs were purified by two consecutive density gradient centrifugations with Histopaque 1077 (7206 g, Ficoll 1.007; Sigma-Aldrich). Finally, RBCs were washed twice with RPMI 2% FBS and were cultured with RPMI 10% FBS, at 14 °C, overnight.

**Figure 5:**
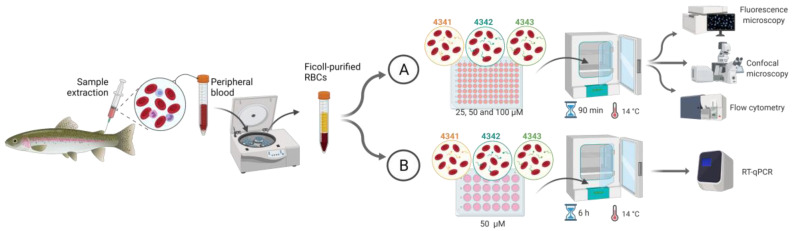
Workflow of in vitro assays in rainbow trout RBCs. (**A**) Evaluation of peptide binding. (**B**) Evaluation of the immune response modulation by the peptides. Created with BioRender.com (accessed on 21 October 2021).

Ficoll-purified RBCs (10^5^ cells/well) were treated with 25, 50, and 100 µM of 4341, 4342, 4343 Rhd-labeled peptides, and incubated for 90 min at 14 °C. Then, RBCs were washed with 200 µL of RPMI 2% FBS and analyzed by flow cytometry, fluorescent microscopy, and confocal microscopy as described below (Figure 5).

To evaluate the possible antiviral immunomodulatory effects of the peptides, ficoll-purified RBCs (10^6^ cells/well) were incubated with 50 µM of 4341, 4342, 4343 Rhd-labeled peptides for 6 h at 14 °C. After that, RBCs were washed, and samples were stored in buffer for RNA extraction and RT-qPCR analysis (Figure 5).

### 4.4. Peripheral Blood Sampling after In Vivo Injection with the Peptides

**Figure 6:**
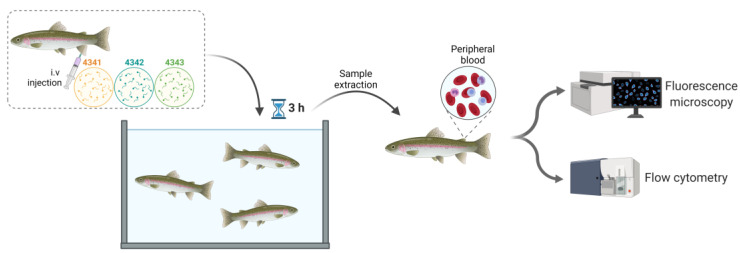
Workflow of in vivo evaluation of peptide binding to rainbow trout RBCs. Created with BioRender.com (accessed on 21 October 2021).

Juvenile rainbow trout (7–10 g) were anesthetized with tricaine methanesulfonate, (Sigma-Aldrich, Madrid, Spain) (40 mg/L) and injected intravenously (i.v) with 100 µM of 4341, 4342, 4343 Rhd-labeled peptides in 50 µL of RMPI 2% FBS using insulin syringes. At 3 hpi, fish were sacrificed by overexposure to tricaine (0.2 g/L), and peripheral blood was extracted and placed on RPMI 2% FBS medium. Then, blood samples were evaluated by means of flow cytometry and fluorescent microscopy as described below (Figure 6).

### 4.5. RNA Isolation and Gene Expression by RT-qPCR

The E.Z.N.A.^®^ Total RNA Kit (Omega Bio-Tek Inc., Norcross, GA, USA) was used for RNA isolation according to the manufacturer’s instructions. Subsequent DNAse treatment was done using TURBO™ DNase (Ambion, Thermo Fisher Scientific, Waltham, MA USA) to eliminate possible residual genomic DNA. RNA was quantified with a NanoDrop Spectrophotometer (Nanodrop Technologies, Wilmington, DE, USA). Then, cDNA synthesis from RNA was performed using M-MLV reverse transcriptase (Invitrogen, Thermo Fisher Scientific) as previously described [65].

To evaluate the gene expression of RBCs treated with 50 µM of the three Rhd-labeled peptides, RT-qPCR was carried out using the QUANTSTUDIO 3 System (Applied Biosystems, Thermo Fisher Scientific Inc.). Gene expression was analyzed by the 2^–ΔΔCt^ method [66]. The *ef1α* gene was used as endogenous control. Primers and probes sequences are listed in Table 3.

### 4.6. Flow Cytometer Assays

RBCs treated in vitro with the Rhd-labeled peptides and blood samples from individuals of rainbow trout injected with the three Rhd-labeled peptides were analyzed by flow cytometry using a FACS Canto II flow cytometer (BD Biosciences, Madrid, Spain). The PE fluorochrome filter was used to detect the Rhd-labeled peptides. A total of 25,000 events were acquired. All the parameters were settled down after recording the suitable control samples. Gating selection for in vitro and in vivo assays are indicated in Supplementary Figures S5 and S6, respectively. Then, the integrated median fluorescence intensity (iMFI) was computed by multiplying the relative frequency (% positive) of cells with the median fluorescence intensity (MFI) of that population (% positive cells × MFI).

### 4.7. Fluorescent Microscopy

In order to track the peptide-cell binding in vitro and in vivo, the three Rhd-labeled peptides were monitored by means of fluorescent microscopy using the IN Cell Analyzer 6000 Cell Imaging system (GE Healthcare, Little Chalfont, UK). For in vitro assays, ficoll-purified RBCs were treated with Rhd-labeled peptides for 90 min, washed, and placed in 96-well plates with RPMI 2% FBS prior to visualization. For in vivo assays, 3 hpi with Rhd-labeled peptides, blood was extracted as described in Section 4.4. Then, cells were placed in 6-well plates with RPMI 2% FBS prior to visualization. dsRed channel was used to visualize Rhd B fluorescence.

### 4.8. Confocal Microscopy

Confocal microscopy was performed to evaluate peptide-RBCs binding in vitro. After 90 min of incubation with Rhd-labeled peptides, RBCs were washed, and nuclei were stained with Hoechst (1 μg/mL) (Sigma-Aldrich) and membrane was stained with CellMask Green (5 μg/mL) (Thermo Fischer Scientific). Images were taken with a Zeiss LSM900 with Airyscan 2 and analyzed with ZEN 3.2 (Blue edition) software (Zeiss, Oberkochen, Germany). Volume rendering was performed using IMARIS Software v9.3 (Bitplane, Zurich, Switzerland).

### 4.9. Software Statistical Analysis

The GraphPad Prism 6 software (GraphPad Software Inc., San Diego, CA, USA) was used for statistical analysis and graphic representation. Flow cytometry data were processed and analyzed using Flowing Software 2.5.1 (www.flowingsoftware.com/ accessed on 30 August 2021). Confocal microscopy images were processed using the ZEN 3.2 (Blue edition) software and analyzed with IMARIS v9.3. Peptide structures were obtained using PEP-FOLD software (https://bioserv.rpbs.univ-paris-diderot.fr/services/PEP-FOLD/ accessed on 22 October 2021) and the images were captured using the program Swiss-Pdb-Viewer (https://www.expasy.org/ accessed on 22 October 2021). Dipole moment calculations were accomplished using BIOVIA Discovery Studio Visualizer (https://discover.3ds.com/ accessed on 22 October 2021). The number of hydrogen bond donor/acceptors was calculated using MarvinSketch software (https://chemaxon.com/products.html accessed on 22 October 2021). LogP calculations were accomplished using ALOGPS 2.1 (http://www.vcclab.org/lab/alogps/ accessed on 22 October 2021).

## Figures and Tables

**Table 1:** Physicochemical and molecular descriptors for each peptide.

Peptide	Dipole Moment (Debye)	N° of Hydrogen Bond Donors	N° of Hydrogen Bond Acceptors	logP
4341	90.132	18	19	−1.73
4342	38.657	26	24	−0.55
4343	42.921	11	10	−2.48

**Table 2:** Peptide sequence of the selected peptides, rhodaminated (Rhd). aas is the abbreviation for amino acid residues.

Name	Peptide Sequence	Length	Source
4341	Rhd-LNKKTVVRKI	10 aas	[43]
4342	Rhd-YASPKCFRYPNGVLACT	17 aas	[44]
4343	Rhd-SPYGRC	6 aas	[45]

**Table 3:** Primers and probes sequences used in quantitative RT-qPCR.

Gene	Forward Primer (5′−3′)	Reverse Primer (5′−3′)	Probe (5′−3′)	Reference or Accession Number
*ef1α*	ACCCTCCTCTTGGTCGTTTC	TGATGACACCAACAGCAACA	GCTGTGCGTGACATGAGGCA	[67]
*ifit5*	CCCTCAATGACTCTGACAAGCA	CCCTGCCCTCATCTTTCTTCT	CCAGCTTCGGCCTGTTTCTGTTCCA	[38]
*mx1-3*	TGAAGCCCAGGATGAAATGG	TGGCAGGTCGATGAGTGTGA	ACCTCATCAGCCTAGAGATTGGCTCCCC	[68]
*nkef*	CGCTGGACTTCACCTTTGTGT	ACCTCACAACCGATCTTCCTAAAC		[28]
*vig1*	CTACAATCAAGGTGGTGAACAATGT	GTGGAAACAAAAACCGCACTTATA	TCTCAAGCTTCGGCAACTCCAAGCA	[28]

## Data Availability

Not applicable.

## Supplementary Materials

The following are available online at https://www.mdpi.com/article/10.3390/ijms222111821/s1.
