# Supplementary figures and images for: Immunomodulatory Lectin-like Peptides for Fish Erythrocytes-Targeting as Potential Antiviral Drug Delivery Platforms
Source: Int J Mol Sci. 2021 Oct 30;22(21):11821. doi: 10.3390/ijms222111821 (PMC8584011; doi:10.3390/ijms222111821)

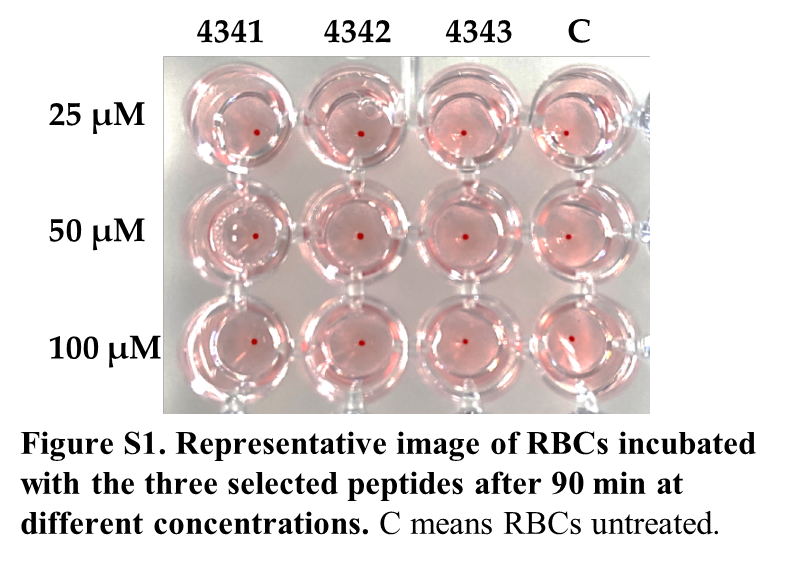

Supplement: Supplementary file 1 [file ijms-22-11821-s001.zip › Figure S1.tif]

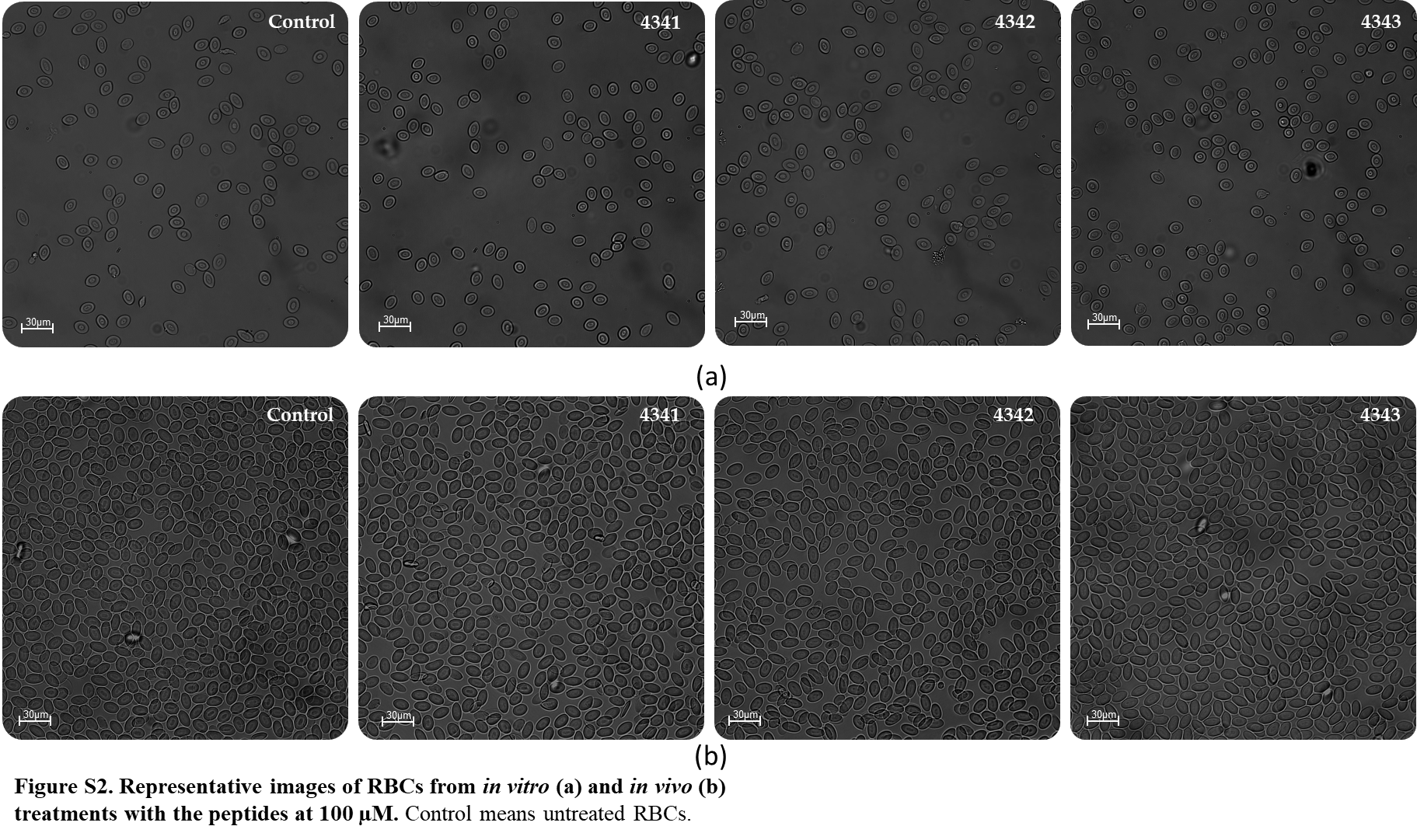

Supplement: Supplementary file 1 [file ijms-22-11821-s001.zip › Figure S2.tif]

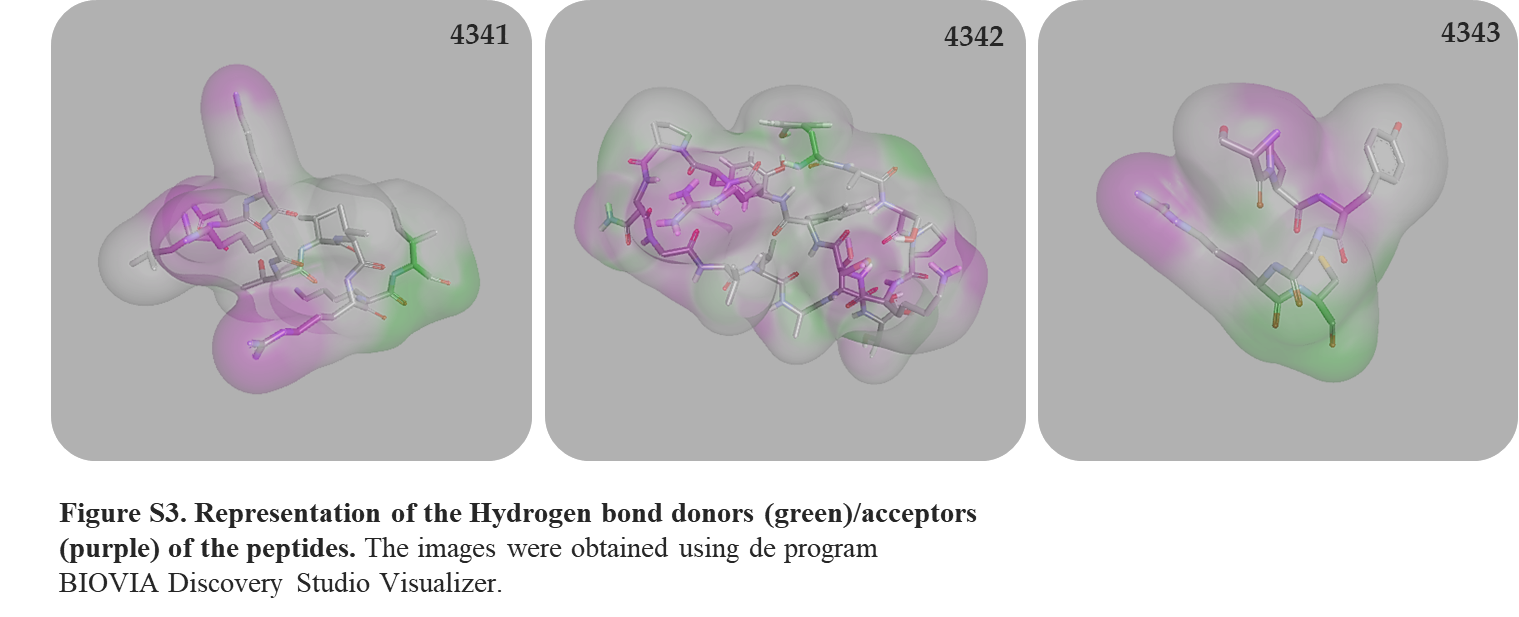

Supplement: Supplementary file 1 [file ijms-22-11821-s001.zip › Figure S3.tif]

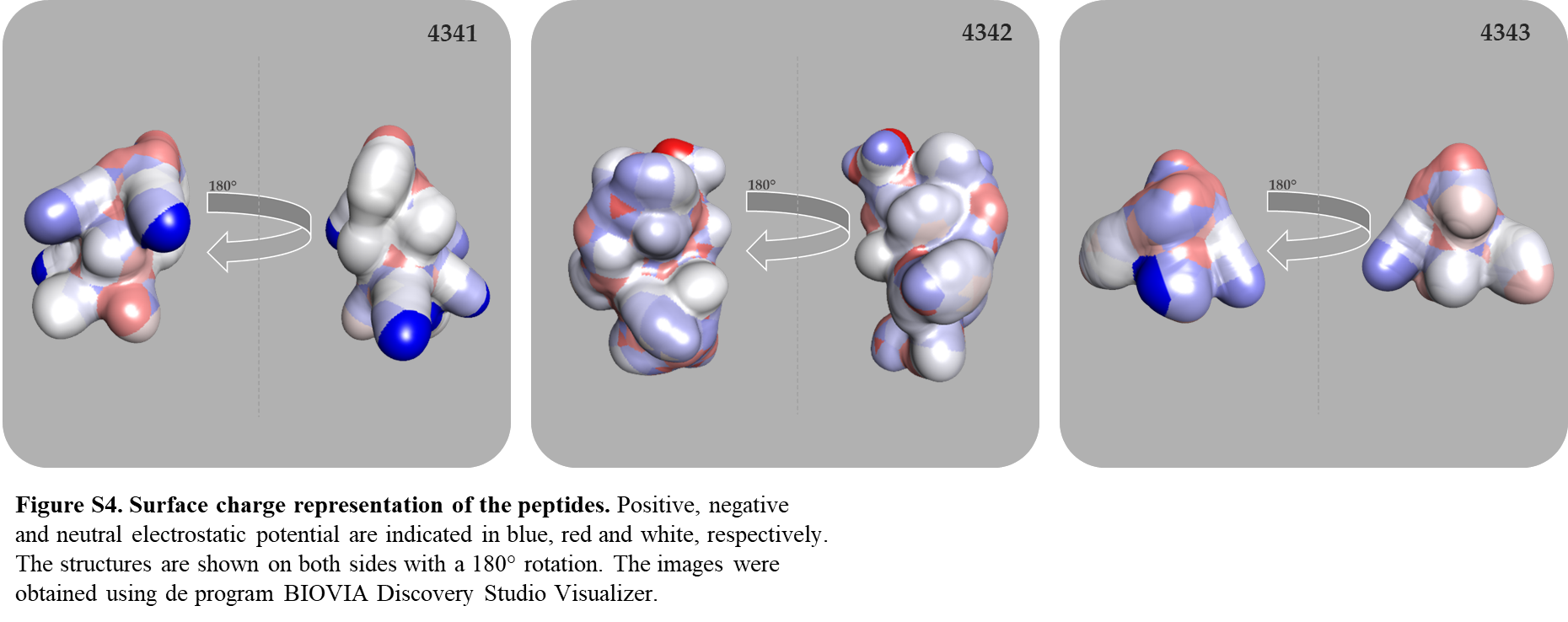

Supplement: Supplementary file 1 [file ijms-22-11821-s001.zip › Figure S4.tif]

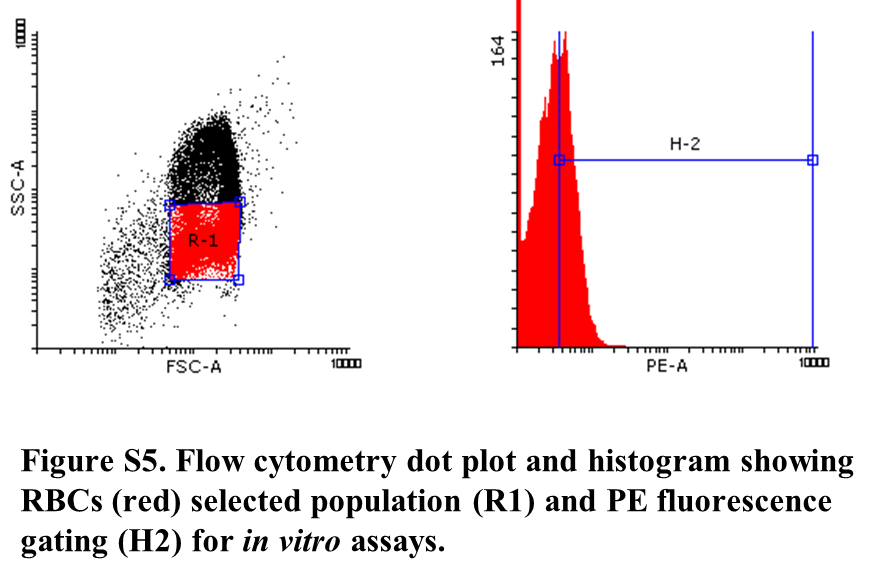

Supplement: Supplementary file 1 [file ijms-22-11821-s001.zip › Figure S5.tif]

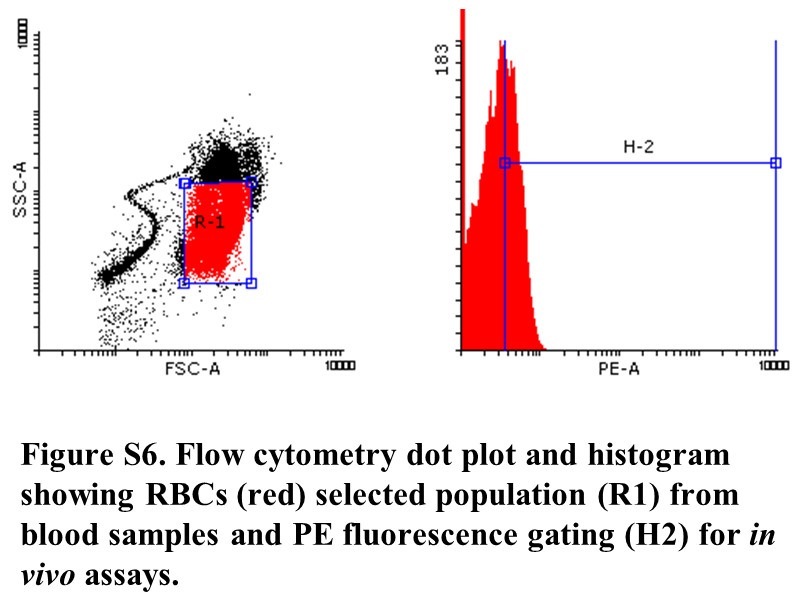

Supplement: Supplementary file 1 [file ijms-22-11821-s001.zip › Figure S6.tif]
